# The efficacy and safety of omega-3 fatty acids on depressive symptoms in perinatal women: a meta-analysis of randomized placebo-controlled trials

**DOI:** 10.1038/s41398-020-00886-3

**Published:** 2020-06-17

**Authors:** Mi-Mi Zhang, Yan Zou, Su-Min Li, Li Wang, Yu-Hui Sun, Le Shi, Lin Lu, Yan-Ping Bao, Su-Xia Li

**Affiliations:** 1 National Institute on Drug Dependence, Peking University, Beijing, 100191 China; 2 Department of Obstetrics and Gynecology, Xiang’ an Hospital of Xiamen University, School of Medicine, Xiamen University, Xiamen, 361102 China; 3 Department of Female Clinical Research, National Research Institute for Family Planning, Beijing, 100081 China; 4 Department of Gynecology, the 305 Hospital of PLA, Beijing, 100017 China; 5 Department of Obstetrics and Gynecology, the First Affiliated Hospital of Harbin Medical University, Harbin, 150001 China; 6 Peking University Sixth Hospital, Peking University Institute of Mental Health, NHC Key Laboratory of Mental Health (Peking University), National Clinical Research Center for Mental Disorders (Peking University Sixth Hospital), Beijing, 100191 China

**Keywords:** Depression, Scientific community

## Abstract

Omega-3 fatty acids (FA), as a nutrient, has been proven effective in major depressive disorder (MDD), however, the results of monotherapy in perinatal depression (PND) remain unclear. To examine the efficacy and safety of omega-3 fatty acids (FA) monotherapy for perinatal depression (PND) compared with placebo. PubMed, Embase, PsycINFO, MEDLINE, Cochrane Library, and CINAHL were searched from inception up to November 2019. The reference lists of relevant review articles and included studies were also reviewed. Randomized placebo-controlled trials examining the efficacy and safety of omega-3 FA monotherapy in perinatal women with depressive symptoms were included. Pooled standard mean differences (SMD) were calculated and random-effects models were adopted for all analyses. Subgroups analyses and meta-regression were performed to quantify characteristics of the subjects and trials influencing the omega-3 response. In addition, meta-regression was conducted to identify the source of heterogeneity. The study protocol was registered at PROSPERO, CRD42020159542. Eight eligible randomized placebo-controlled trials were included involving 638 participants. There was a significant effect of omega-3 FA on perinatal depression. Omega-3 with higher ratio of EPA/DHA (≥1.5) had significant efficacy both in mild-to-moderate pregnant and postpartum depression with low incidence of side effects. Among the included trials reporting adverse effects, there was no significant difference in incidence of gastrointestinal and neurologic events between the omega-3 and placebo groups. There was no evidence of publication bias. Our findings suggested that omega-3 FA significantly improved depressive symptoms in perinatal women regardless of pregnant or postpartum and well-tolerated. Furthermore, the omega-3 response was linked to higher EPA proportion in omega-3 formula and mild- to-moderate depression.

## Introduction

Perinatal depression (PND) is normally defined as onset of a depression episode, ranging from mild to severe, during pregnancy or postpartum within 1 year after delivery. Depression disorder during perinatal period among childbearing-age women is increasingly common worldwide, with a prevalence of 11.9% in general and higher risk in low-income countries, as high as 25% antepartum and 20% postpartum^1,2^. Different from general “maternity blues”, with remission in 1 or 2 weeks; PND symptoms are more severe and persistent if they are ignored^3^. Symptoms of PND include depressed mood, marked lack of interest or pleasure in all or most activities, significant weight loss or gain, insomnia or excessive sleep, psychomotor excitement or retardation, fatigue or poor energy, feelings of meaninglessness or guilt, impaired thinking or concentration, and the risk of self-harm or even suicide^4,5^. Moreover, babies’ health can be adversely affected by PND. Depression during pregnancy without treatment can lead to poor pregnancy outcomes, such as small in size, preterm birth, and the risk for low birth weight^6–8^. In the long-term, offspring of mothers subjected to PND are prone to defects in cognitive development in childhood, have a higher risk to display depressive behavior in adolescence and be involved in criminal activities in adulthood^9,10^.

The treatment of PND is a clinical dilemma. Almost all antidepressants can cross the placenta more or less. Almost all of pregnant women refuse to take medications with fear of potential side effects on offspring, for example major cardiovascular malformations and poor neonatal adaptation syndromes^11^. Omega-3 FA, mainly comprised of docosahexaenoic acid (DHA) and eicosatetraenoic acid (EPA), is a type of polyunsaturated FA (PUFA). DHA constitutes an essential part of cell membranes and is found to be abundant in brains and retina^12^. Both DHA and EPA are precursors of a group of eicosanoids, which have anti-inflammatory effects^13,14^. Naturally, human is unable to synthesize omega-3 FA in vivo, hence regular dietary intake of deep-sea fish which is enriched in omega-3 is essential for health. As a kind of indispensable nutrient substance, plenty of studies have verified the moderate beneficial effects of omega-3 (monotherapy or adjuvant) on some mental diseases like major depressive disorder (MDD)^15,16^. The supposed mechanisms are anti-inflammatory and anti-oxidant. Therefore, considering of safety in perinatal period, omega-3 FA are supposed to be a promising therapy alternative for PND.

A previous study^17^ showed that three dose groups of omega-3 FA had achieved significant changes of depressive symptoms from baseline and well-tolerated for postpartum depression. However, the study had the disadvantage of showing no statistically significant difference in scores of depression scales among groups and no placebo group. In the following year, there had published several RCTs evaluating whether DHA and EPA monotherapy had a superiority over placebo. The results were mixed and sample size in each study is small^18–20^. A previous meta-analysis^21^, in which subjects with PND were mixed with healthy gestational women, showed no beneficial effect of omega-3 PUFA over placebo on perinatal depression. Next, another meta-analysis^22^, in which only four RCTs were available, showed that omega-3 FA monotherapy was superior over placebo for PND.

Base on above-mentioned conflicting results, the aim of this meta-analysis is to provide the convincing evidence whether omega-3 FA monotherapy can exert a positive effect on PND. The clinical manifestations of perinatal depression are similar to those of other periods of depression. For the evaluation of clinical symptom severity, other evaluation scales applicable to depression are also applicable to the evaluation of perinatal depression, in addition to those specifically used for the evaluation of perinatal depression. Therefore, according to the included published papers actually used the evaluation scale, the Edinburgh Postnatal Depression Scale (EPDS) was preferred choice to measure depressive symptoms in perinatal women in our meta-analysis. When EPDS was not applied in studies, Hamilton Depression Rating Scale (HAMD) or Beck Depression Inventory (BDI) was used instead. Subgroup analyses were conducted according to the constituent of omega-3 FA, intervention duration, and baseline depression severity and pregnant or postpartum. Meanwhile, the safety of fish oil such as the side effects and tolerability were also evaluated. The conclusions are expected to offer an alternative therapy for PND and bring light for women suffering from PND and their babies.

## Methods

### Search strategy

The Preferred Reporting Items for Systematic Reviews and Meta-analyses (PRISMA) statement guidelines were followed^23^. The study protocol for this meta-analysis was registered on the International Prospective Register for Systematic reviews (PROSPERO identifier: CRD42020159542). PubMed, Embase, PsycINFO, MEDLINE, Cochrane Library, and CINAHL from inception up to November 2019 were searched by two independent researchers. The search terms used for the literature search were (omega-3 FA or DHA or EPA or fish oil) and (pregnant or perinatal or prenatal or postpartum) and depression, without language limitations. There were also no restrictions regarding sample size and study period. The titles and abstracts of the found studies were screened to determine the potential eligibility. In addition, the references of selected articles and related reviews were examined for extra available studies not included in above databases. In case of disagreement for eligibility, we discussed in detail according to inclusion criteria to reach consensus.

### Inclusion criteria

According to PICOS (Participants, Interventions, Comparisons, Outcomes, Study design) principle, the included studies should fulfill the following inclusion criteria, Participants (P): pregnant or postpartum women with major depressive disorder diagnosed according to DSM-IV or mild and above depression symptoms determined by relevant scales. Studies examining the prophylactic effects of fish oils on depressive symptoms among health perinatal women were excluded. Intervention (I): omega-3 FA including DHA and EPA as monotherapy. The dosage and duration of omega-3 should be clearly stated. Comparison (C): omega-3 group vs placebo group. Outcome (O): efficacy and safety. The levels of depressive symptoms at baseline and endpoint of treatment both in omega-3 and placebo groups based on standard depression rating scales. Safety evaluation including incidence of adverse events in both groups. Study designs (S): randomized double- or triple-blinded placebo-controlled trials. Systematic reviews, meta-analyses, case reports or series, no placebo trials and open-label trials are excluded. When studies have overlapping data, we only included the study with the largest sample size among them.

### Data extraction

Data extraction was based on intent to treat (ITT) analysis or modified ITT data (i.e., at least one dose or at least one follows-up assessment) if provided. Two independent reviewers performed data extraction. Any discrepancy was discussed in depth until consensus achieved. When necessary, we contacted the study investigators for detailed information. Relevant data on subjects’ demographic characteristics, baseline depression severity, rating scales, dosage and component of omega-3 FA, treatment duration and outcome measurements including scale scores at baseline and endpoint were extracted. Meanwhile, we extracted information about adherence assessment and side effects on mothers or babies. Preferred clinical rating scale was Edinburgh Postnatal Depression Scale (EPDS), for it was designed for perinatal population. In case of EPDS not available, Hamilton Depression Rating Scale (HDRS) or Beck Depression Inventory (BDI) was used instead. Non-English language studies such as Persian were translated by Google Translation.

### Assessment of study quality

Two trained reviewers independently assessed the study quality and risk of bias for each study according to the Cochrane Handbook for Systematic Reviews of Interventions^24^. The evaluated component was based on six domains: random sequence generation, allocation concealment, adequacy of blinding (including investigators, participants, and statistical analysts), attrition (completeness of outcome data and the use of intention-to-treat analysis), selectively reporting, and other biases (conflict of interests, baseline imbalance). We judged each potential source of bias as high, low or unclear bias. All disagreements were resolved by discussion.

### Statistical analysis

The mean improvements of depressive symptoms in terms of psychometric scales on omega-3 FA group and placebo group were compared as primary analyses to measure the omega-3 efficacy. Standard mean difference (SMD) was chosen as summary statistic for continuous data in this meta-analysis and calculated by pooling the standardized mean improvement of each study. SMD has an advantage over weighed mean difference in case of different scales or measurements utilized in studies. In line with conventional interpretations, SMDs were classified as negligible (<0.2), small (0.2–0.4), moderate (0.4–0.8), or large (>0.8). If SDs were not available, we extracted other statistical parameters reported in the study to estimate them. Considering potential heterogeneity, we used a random-effects model. Heterogeneity assessment was performed by calculated Q-statistic. *I*^2^ statistic was used to quantify heterogeneity, while the value higher than 50% indicated significant heterogeneity. Publication bias was evaluated by funnel plot and Egger’s test. We conducted sensitivity analyses to confirm the stability of our conclusions.

In view of potentially high heterogeneity in previous studies, we performed subgroup analyses and meta-regression as secondary analyses. For subgroup analyses, the studies were stratified based on (1) EPA and DHA proportion: the ratio of EPA/DHA ≥ 1.5 and <1.5; (2) pregnant and postpartum; (3) baseline depression severity: mild, moderate to severe; (4) intervention duration: <8 weeks and ≥8 weeks. With regard to classifying baseline depression severity, we chose the cut-offs for EPDS (mild: 10–12; moderate: 13–15; severe: ≥16). The depression severity base on other rating scales were converted to the EPDS according to previously defined algorithms^25^.

A random-effects meta-regression was conducted to investigate potential sources of variability between studies, as it took inherent study-to-study variability into account. We conducted meta-regressions by risk of bias score and study design. To be specific, the variables included: sample size, scales, and quality score. A significance threshold of *P* < 0.05 indicated the contribution to heterogeneity.

All statistical analyses were performed using Stata 12.0 (StataCorp, College Station, TX, USA) and RevMan 5.3 (Cochrane Collaboration, Copenhagen, Denmark).

## Results

### Selection of studies

**Fig. 1:**
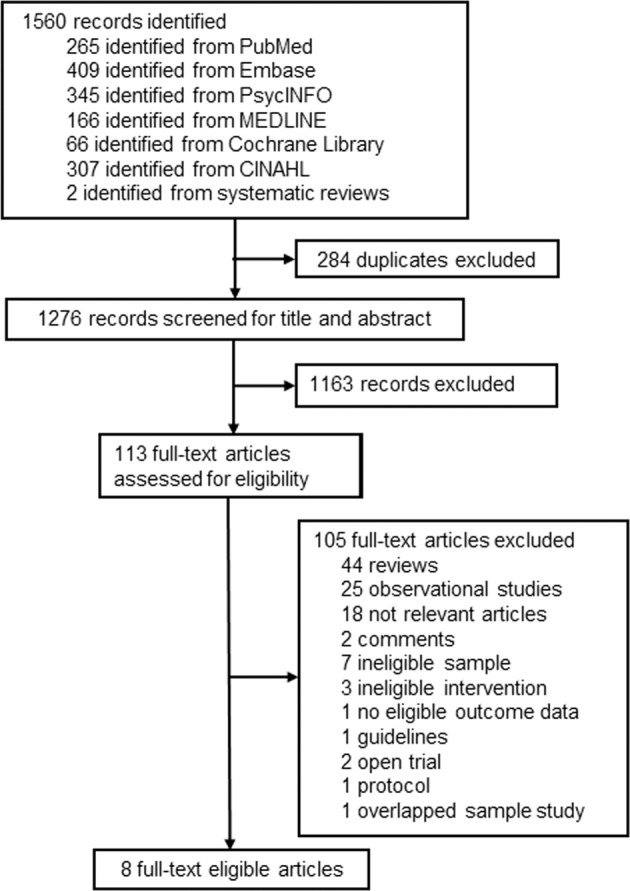
The flowchart describing the process of study inclusion.

The flowchart of literature search was presented in Fig. 1. A total of 1560 records were obtained. Our primary search strategy from above electronic databases resulted in 1558 articles, including 265 records in PubMed, 409 records in Embase, 345 records in PsycINFO, 166 records in MEDLINE, 66 records in Cochrane Library, and 307 records in CINAHL. In addition, 2 records^26,27^ were identified through references of related systematic reviews and included studies. After 284 duplicated studies being removed, there remained 1276 records. Then titles and abstracts of studies were examined, 1163 of which were excluded for not related to omega-3 FA and perinatal depression. We carefully reviewed 113 articles for full text. Of these, 105 studies were excluded for either not meeting inclusion criteria or overlapping samples with included studies, and 8 eligible studies^18–20,28–30^ were identified. Of the 8 eligible trials, there were two Persian trials^26,27^ which were identified through a relevant systematic review^31^ and an included study^28^. The content of these two studies was translated by Google translation. Finally, we included eight randomized double- or triple-blinded placebo-controlled trials evaluating the efficacy of omega-3 FA monotherapy in perinatal depression in our meta-analysis.

### Characteristics of included studies

The basic characteristics of eight eligible studies were displayed in Table 1. In these eligible studies, four^18–20,29^ used several measuring scales including EPDS, three^26–28^ used BDI, and one^30^ used HDRS. In terms of diagnosis methods, in three of the studies, MDD was diagnosed by using the formal DSM-IV diagnostic criteria^18–20^ and in other studies, mild-to-moderate depressive symptoms were diagnosed by rating scales. With regard to pregnant or postpartum depression, two^18,20^ of eight trials evaluated both pregnant and postpartum depression, four^19,28–30^ of eight trials evaluated only pregnant depression, and two^26,27^ of eight trials evaluated only postpartum depression. Only one trial^28^ was targeted for primiparous women. Four trials^26–29^ were conducted in Iran, one^20^ in America, one^18^ in Australia, one^19^ in Taiwan, China, and one^30^ both in Japan and Taiwan, China. The oral dosage of omega-3 differed from 1 to 6 g/day. Two trials^26,28^ in Iran did not mention the EPA and DHA ratio in omega-3 FA. One study^19^ in Taiwan, China performed a 1-week lead-in trial to exclude placebo responders. One study^20^ added supportive psychotherapy for ethical reason.

**Table 1:** Characteristics of included studies.

Study	Country	Clinical group	Number (ω-3 FA/placebo)	Blinding	Daily dosage	Pregnant or postnatal	Treatment duration (weeks)	Baseline depression severity	Rating scales
Rees et al.^18^	Australia	Major depression	26 (13/13)	Double blind	6 g (27.3% DHA, 6.9% EPA)	Perinatal	6	Severe	EPDS, HRSD, BDI
Freeman et al.^20^	USA	Major depression	51 (28/23)	Double blind	1.9 g (1.1 g EPA, 0.8 g DHA)	Perinatal	8	Severe	EPDS, HRSD, BDI
Su et al.^19^	Taiwan	Major depression	33 (17/16)	Double blind	2.2 g EPA, 1.2 g DHA	Pregnant	8	Severe	EPDS, HRSD, BDI
Ivanbaga et al.^26^	Iran	Mild-to- moderate depression	120 (60/60)	Double blind	1 g omega-3	Postnatal	8	Moderate	BDI
Nahidi et al.^27^	Iran	Depression	70 (35/35)	Double blind	1 g omega-3 (120 mg DHA, 180 mg EPA)	Postnatal	4	Mild	BDI
Kaviani et al.^28^	Iran	Mild depression	80 (40/40)	Double blind	1 g omega-3	Pregnant	6	Mild	BDI
Farshbaf-Khalili et al.^29^	Iran	Depression (EPDS < 20)	150 (75/75)	Triple blind	1 g omega-3 (120 mg DHA, 180 mg EPA)	Pregnant	14	Mild	EPDS
Nishi et al.^30^	Japan, Taiwan	Depression (EPDS ≥ 9)	108 (55/53)	Double blind	1206 mg EPA, 609 mg DHA	Pregnant	12	Mild	HRSD

*Ω-3 FA* Omega-3 fatty acids, *EPDS* Edinburgh Postnatal Depression Scale, *BDI* Beck Depression Inventory, *HRSD* Hamilton Rating Scale for Depression.

### Quality assessment

The summary of risk of bias assessment was shown in sTable 1 in the supplement. The overall quality of included study was moderate. The detailed information about blinding and allocation concealment was insufficient in most studies. All trials’ statistical analyses were based on intention-to-treat or modified intention-to-treat methods. Two trials^18,20^ had a relatively high risk of attrition bias, in which the drop-out rate was higher than 15%. All trials conducted adherence assessment and the results were favorable.

### Omega-3 efficacy in perinatal depression

**Fig. 2:**
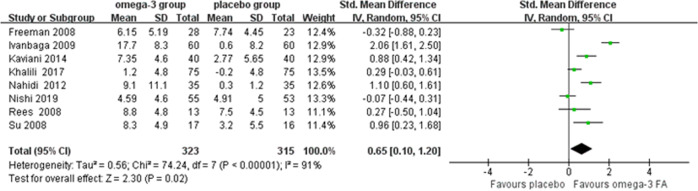
Forest plot of treatment of omega-3 FA in perinatal depression. SD, standard deviation; Std. Mean difference, standardized mean difference; IV. Random, inverse variance heterogeneity; CI, confidence interval.

Eight trials including 638 subjects were included in this meta-analysis. The results were based on EPDS in four studies^18–20,29^, BDI in three studies^26–28^, and 17-item HDRS in one study^30^. Overall, omega-3 FA monotherapy during perinatal period had moderate effects on reducing depressive symptoms in perinatal women compared with placebo (SMD = 0.65, 95% confidence interval (CI): 0.10, 1.20, *P* = 0.02) (Fig. 2). However, high heterogeneity between studies was detected (*I*^2^ = 91%).

#### Subgroup analyses and meta-regression

**Fig. 3:**
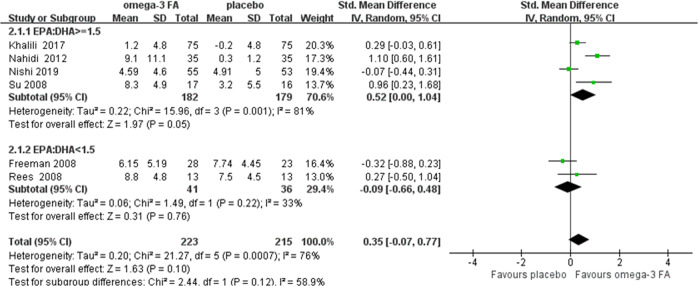
Forest plot of treatment effects of omega-3 FA for perinatal depression, separated by the ratio of EPA/DHA in omega-3, the ratio ≥1.5 or <1.5. Two trials did not mention the EPA and DHA content and were not included. SD, standard deviation; Std. Mean difference, standardized mean difference; IV. Random, inverse variance heterogeneity; CI, confidence interval.

**Fig. 4:**
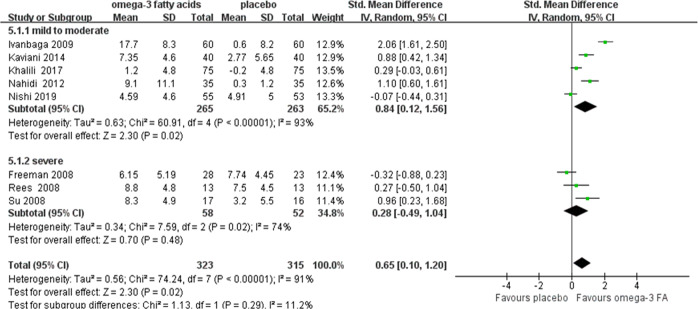
Forest plot of treatment effects of omega-3 FA for perinatal depression, separated by baseline depression severity, mild, moderate to severe. SD, standard deviation; Std. Mean difference, standardized mean difference; IV. Random, inverse variance heterogeneity; CI, confidence interval.

To examine whether omega-3 FA had discrepant efficacy in reducing depressive symptoms between pregnant and postpartum state, we performed subgroup analysis to synthesize results of pregnant and postpartum depression, respectively. Two trials examined perinatal state and were not included^18,20^. As a result, omega-3 FA had beneficial effects on both pregnant depression (SMD = 0.46, 95% confidence interval (CI): 0.01, 0.91, *P* = 0.05) and postpartum depression (SMD = 1.59, 95% confidence interval (CI): 0.65, 2.53, *P* < 0.001) compared with placebo (sFig. 2 in the supplement). Furthermore, to investigate whether the EPA proportion played a key role in the efficacy, we classified the ratio of EPA and DHA as ≥1.5 or <1.5. Two trials did not mention the ratio of EPA/DHA and thus were excluded^26,28^. In ratio ≥1.5 group, the effects of omega-3 FA approached statistical significance compared with placebo (SMD = 0.52, 95% confidence interval (CI): 0.00, 1.04, *P* = 0.05), and no significant effects were demonstrated in the ratio <1.5 group (SMD = −0.09, *P* = 0.76) (Fig. 3). According to the depression severity at baseline, omega-3 had a moderate to large significant effects on participants with mild-to-moderate depression compared with placebo (SMD = 0.84, 95% confidence interval (CI): 0.12, 1.56, *P* = 0.02). That was not the case in the severe depression group (SMD = 0.28, *P* = 0.48) (Fig. 4). In terms of intervention period, omega-3 demonstrated remarkably beneficial effects on the group of less than 8 weeks (SMD = 0.84, 95% confidence interval (CI): 0.43, 1.24, *P* < 0.0001), and no superiority was shown in the group of 8 weeks or longer compared with placebo (SMD = 0.58, *P* = 0.17) (sFig. 3 in the supplement). Meta-regression was conducted to identify sources of heterogeneity. The results demonstrated no significant difference in any covariate analysis.

### Publication bias and sensitivity analyses

The funnel plot (sFig. 1 in the supplement) appeared symmetrical and Egger’s regression test suggested no significant publication bias (*t* = 0.38, *P* = 0.718). Sensitivity analyses using a random-effects model indicated the significant effects of omega-3 FA were not affected by any single study.

### Safety and tolerability

Overall, the dosages of omega-3 in included studies varied from 1 to 6 g per day and were all well-tolerated for most subjects. Although drop-out rates were more than 15% in two studies, no participants discontinued for side effects. Among eight included trials that reported adverse effects in this meta-analysis, there were not significant differences in incidence of side effects between two groups in each trial. To sum up, the most commonly reported adverse effects were uncomfortable symptoms of digestive tracts, including nausea, vomiting, and increased stool frequency. Other non-specific neurologic symptoms included dizziness, fatigue, and insomnia. The reported events were mostly mild and self-limited. No adverse effects regarding babies were mentioned in all studies and were not able to be evaluated.

## Discussion

This extensive meta-analysis on 638 participants, including 323 participants in the omega-3 FA monotherapy group and 315 participants in the placebo group, shows that omega-3 FA has a superior antidepressant effect over placebo on PND with a moderate effect size. Notably, subgroup analyses showed omega-3 monotherapy has significant beneficial effects on both pregnant and postpartum depression. The effects on PND were associated with higher ratio of EPA in omega-3, shorter intervention period and mild-to-moderate depression. Omega-3 seems to be generally safe and well-tolerated.

The exact mechanism through which omega-3 improve the depressive symptoms in perinatal women remains unclear. During perinatal period, omega-3 FA mainly DHA is in higher demand for women, because they are constantly transmitted from moms to babies for brain development and retina maturation either through placenta or breast feeding^12,32^. Hence there are higher risks for mothers of deficiency in omega-3 without timely and proper supplementation. As anti-inflammatory substance in nature, a lack of omega-3 predispose perinatal women to suffer from inflammation-related disease like depression^33,34^. Indeed, Lin et al.^35^ found women with PND had a lower level of DHA, but not EPA in meta-analysis. In fact, both EPA and DHA decreased in prenatal depression.

Considering the physiology differences between pregnancy and postpartum periods, the specific mechanism underlying pregnant and postpartum depression may be distinct. Therefore, we evaluate the effects of omega-3 on prenatal and postnatal depression, separately. Consequently, the effects are significant in both, and more obvious in postpartum depression (PPD). Of note, the only two trials of PPD were all conducted in Iran and the results may not be representative and should be taken cautiously. To achieve more comprehensive and convincing proof, larger sample size and multi-center clinical trials are needed for validation.

Besides, subgroup analysis revealed better response relied on higher ratio of EPA/DHA (≥1.5) in line with previous meta-analyses results in general MDD^15,16,36^. Based on the results, omega-3 exert an antidepressant efficacy in PND due to the anti-inflammation of EPA rather than addition of DHA to membrane. With regard to anti-inflammatory effects, EPA, rather than DHA plays a major role. First of all, EPA can decrease the production of pro-inflammatory cytokines, TNF-α, IL-1β, IL-6, IL-8, and interferon-γ (IFN-γ), which have a close association with depression^37^. Furthermore, the product of EPA, (Leucocyte triene B5) LTB5, is a competitive antagonist to highly pro-inflammatory LTB4 derived from arachidonic acid (AA)^38^. In addition to anti-inflammation, EPA is a natural ligand to the peroxisome proliferator-activated receptor gamma (PPARγ) nuclear transcription receptor, which can prevent the expression of nuclear factor-kappa B (Nf-kB) and inhibit the activation of the signaling pathway related to pathophysiology of depression^39^. However, confined by the number of included studies in this meta-analysis, the most efficient ratio of EPA/DHA should be explored in the future research.

The subjects in eight eligible RCTs varied in baseline depression severity, and we wanted to explore whether it would determine omega-3 response. Firth et al.^15^ mentioned in their meta-review that omega-3 FA had a small positive effect on reducing depressive symptoms in general individuals with indicated depression (no diagnosis of MDD). The group of mild-to-moderate PND in our meta-analysis was also determined by rating scales rather than diagnosed as MDD by clinical criteria. Consistent with Firth’s study, we obtained a significant benefit of omega-3 in degree of mild to moderate. However, the discrepant results appeared regarding the severity degree or MDD group. Of the three related trials, two concluded no significant effects. High DHA concentration and extra psychotherapy may cover up the effects of omega-3. In addition, compared with mild-to-moderate group, the sample size is relatively small in severe group and the large-scale studies aimed at severe depression population are in need to be carried out.

As for the results of intervention duration subgroup analysis, better therapeutic effects of omega-3 were associated with shorter duration (<8 weeks), which is in line with the meta-regression results of duration in a previous meta-analysis about omega-3 efficacy in major depression disorder^40^. On the one hand, we noticed the two studies in group of less than 8 weeks with positive results were performed in Iran. On the other hand, from the point view of the whole study, the duration of trials indicating the advantage of omega-3 treatment over placebo ranged from 4 to 14 weeks. Thus, we speculated the intervention period may not a key factor to determine the omega-3 efficacy. Nevertheless, the speculation deserves to be proved in future studies.

Besides, all four trials in Iran had positive effects of omega-3 on PND compared with placebo. We found that the subjects in three studies being mildly depressed and subjects in 1 study being moderately depressed. In view of the relationship, future study testing the omega-3 efficacy in PND should take the severity of depression at baseline into consideration and divide groups according to it.

Perinatal depression is an umbrella term for a group of heterogeneous entities^41,42^. Different patients present different symptom dimensions, including depressed mood, anhedonia, and anxiety, and thus the corresponding therapy should also be personalized. Future studies can categorize participants into similar symptom domains according to EPDS and test which domain has the best omega-3 response. Moreover, it is necessary to distinguish the onset of depressive symptoms is during perinatal period or before, for the underlying etiology may be different between. Of note, we found higher EPA proportions in omega-3 formula led to better treatment effects in this meta-analysis. It has been demonstrated that inflammation is a common mechanism for MDD^43,44^ and EPA has anti-inflammatory effects. Future clinical trials about omega-3 efficacy in PND can measure the inflammation levels in blood at baseline and evaluate whether it will predict the omega-3 efficacy. After all, the definite mechanism of omega-3 efficacy and development of PND remained unknown, the proposals are expected to provide targets for future precision medicine.

### Limitations and strengths

The present study has some limitations. First, a high heterogeneity exists in our meta-analysis. Subgroup analyses and meta-regression still cannot find the source of heterogeneity. Second, the number of included studies and sample size are relatively small, which lead to a wide 95% confidence interval. Third, the dosage kinds of omega-3 are not enough to perform a subgroup analysis. So, we are unable to suggest an appropriate dosage range.

### Conclusions

Our findings indicating the superiority of omega-3 over placebo updated evidence for the debate of the efficacy of omega-3 FA on PND and provide strong foundation for future clinical trials to guide the omega-3 application in PND. However, restricted by current include studies, more studies with big sample size and high-quality RCTs are required in the future to verify the conclusions.

## Supplementary information

Supplementary Material

Supplementary eFig. 1

Supplementary eFig. 2

Supplementary eFig. 3
