# Supplementary material for: The efficacy and safety of omega-3 fatty acids on depressive symptoms in perinatal women: a meta-analysis of randomized placebo-controlled trials
Source: Transl Psychiatry. 2020 Jun 17;10:193. doi: 10.1038/s41398-020-00886-3 (PMC7299975; doi:10.1038/s41398-020-00886-3)
Supplement: Supplementary file 1 — Supplementary Material [file 41398_2020_886_MOESM1_ESM.docx]

**Supplementary material**

**The efficacy and safety of omega-3 fatty acids on depressive symptoms in perinatal women: A meta - analysis of randomized placebo-controlled Trials**

Mi-Mi Zhang^1, 2$^, Yan Zou^3$^, Su-Min Li^4^, Li Wang^4^, Yu-Hui Sun^5^, Le Shi^6^, Lin Lu^1, 6^, Yan-Ping Bao^1*^, Su-Xia Li^1*^

^1.^ National Institute on Drug Dependence, Peking University, Beijing 100191, China

^2.^ Department of Obstetrics and Gynecology, Xiang’ an Hospital of Xiamen University, School of Medicine, Xiamen University, Xiamen 361102, China

^3.^ Department of Female Clinical Research, National Research Institute for Family Planning, Beijing 100081, China

^4.^ Department of Gynecology，305 Hospital of PLA, Beijing 100017, China

^5.^ Department of Obstetrics and Gynecology, the First Affiliated Hospital of Harbin Medical University, Harbin 150001, China

^6.^ Peking University Sixth Hospital, Peking University Institute of Mental Health, NHC Key Laboratory of Mental Health (Peking University), National Clinical Research Center for Mental Disorders (Peking University Sixth Hospital), Beijing 100191, China

**Short Title:** Effects of omega-3 fatty acids on perinatal depression

Abstract: 275 words

Introduction: 706 words

Discussion: 1141 words

Total text: 4171 words

Figures: 4

Tables: 1

$ Equally contribute to this work

***Corresponding author**

Su-Xia Li, MD, PhD

National Institute on Drug Dependence, Peking University

38 Xue Yuan Road, Haidian District, Beijing 100191, China

Tel: +86-10-82802470 ext. 313

Fax: +86-10-62032624

E-mail: [li313@bjmu.edu.cn](mailto:li313@bjmu.edu.cn)

And

Yan-Ping Bao, PhD

National Institute on Drug Dependence, Peking University

38 Xue Yuan Road, Haidian District, Beijing 100191, China

Tel: +86-10-82802470 ext. 412

Fax: +86-10-62032624

E-mail: [baoyp@bjmu.edu.cn](mailto:baoyp@bjmu.edu.cn)

**Acknowledgments**

This work was supported in part by the National Natural Science Foundation of China (no. 81871071 and 81171251) and Beijing Municipal Natural Science Foundation (no. 7162101).

**eTable 1. Risk of Bias**

+ : Low risk of bias, - : High risk of bias, ? : Unclear risk of bias

**eFigure Legends**

**eFig 1.** Funnel plot of effect sizes for clinical trials included in the meta-analysis. Published trials are depicted as dark circles.

**eFig. 2.** Forest plot of treatment effects of omega-3 FA for perinatal depression, separated by pregnant or postnatal. Two trials testing perinatal depression were not included. SD, standard deviation;Std. Mean difference, standardized mean difference; IV. Random, inverse variance heterogeneity; CI confidence interval.

**eFig. 3.** Forest plot of treatment effects of omega-3 fatty acids for perinatal depression, separated by intervention duration,< 8 weeks or ≥8 weeks. SD, standard deviation; Std. Mean difference, standardized mean difference; IV. Random, inverse variance heterogeneity; CI, confidence interval.
