# Supplementary figures and images for: The efficacy and safety of omega-3 fatty acids on depressive symptoms in perinatal women: a meta-analysis of randomized placebo-controlled trials
Source: Transl Psychiatry. 2020 Jun 17;10:193. doi: 10.1038/s41398-020-00886-3 (PMC7299975; doi:10.1038/s41398-020-00886-3)

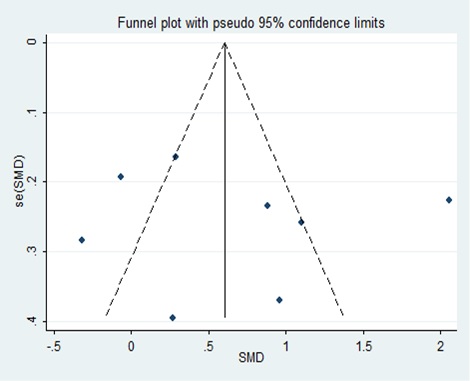

Supplement: Supplementary file 2 — Supplementary eFig. 1 [file 41398_2020_886_MOESM2_ESM.jpg]

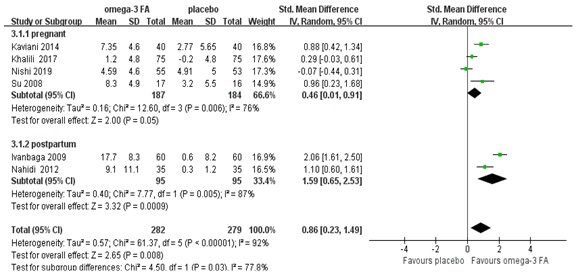

Supplement: Supplementary file 3 — Supplementary eFig. 2 [file 41398_2020_886_MOESM3_ESM.jpg]

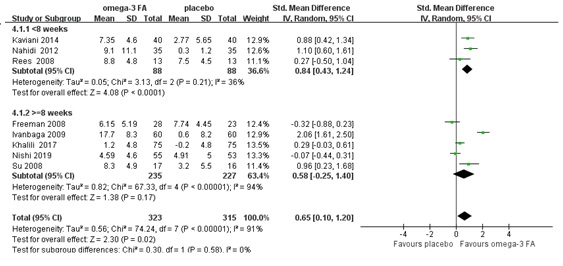

Supplement: Supplementary file 4 — Supplementary eFig. 3 [file 41398_2020_886_MOESM4_ESM.jpg]
